# Virtual Reality Training for Pediatric Sedation Challenges: A Cross-Sectional Study

**DOI:** 10.7759/cureus.79423

**Published:** 2025-02-21

**Authors:** Nadia Shaikh, Fiona S Gruzmark, Francesca Rios, Curie Subramanian, Keith Hanson, Teresa Riech

**Affiliations:** 1 Department of Pediatrics, OSF Saint Francis Medical Center, Peoria, USA; 2 Department of Pediatrics, University of Illinois College of Medicine, Peoria, USA; 3 Department of Emergency Medicine, University of Miami, Jackson Health System, Miami, USA; 4 Department of Pediatrics, Washington University School of Medicine in St. Louis, St. Louis, USA; 5 Department of Hospital Medicine, OSF Healthcare Children's Hospital of Illinois, Peoria, USA; 6 Department of Pediatric Emergency Medicine, OSF Healthcare Children's Hospital of Illinois, Peoria, USA

**Keywords:** airway emergency, medical simulation, medical training, pediatric sedation, virtual reality

## Abstract

Background

Teaching the management of pediatric sedation-related airway concerns is challenging due to the time-sensitive interventions required and their infrequent occurrence. Recognizing this educational gap in the sedation experience, virtual reality (VR) was utilized to prepare learners and provide an immersive training experience in a controlled environment.

Methods

We describe simulation-based training using a VR environment, Enduvo Inc., an innovative software for medical trainees. This two-hour course creates a problem-based learning experience, allowing learners to apply their knowledge and develop a framework for managing these stressful and less commonly encountered situations.

Results

From 2019 to 2022, 35 learners participated in the sedation course. After completing the VR modules, all learners completed a blinded survey on their experience and preparation for sedation-related situations. Based on our initial data, self-reported confidence and preparedness in handling complex situations during pediatric sedation rated high, with an overall response rate of 100%. Learners reported improved confidence and self-preparedness after completing the modules.

Conclusion

VR for pediatric sedation training creates a distinctive learning environment due to its immersive nature and has been well-received. VR could be an effective method for educating residents and physicians in this specialized field.

## Introduction

Mastering the skill of pediatric procedural sedation is a complex yet essential skill for pediatric trainees. Understanding how to manage sedated pediatric patients, grasping the nuances of indications and contraindications, recognizing complications, and being prepared for safe procedural sedation can be challenging [1,2]. Many outpatient procedures that were previously performed under anesthesia are now frequently done outside the operating room with varying levels of sedation by subspecialists, particularly intensivists and pediatric hospitalists. Thus, there is a growing need for this training despite a limited number of dedicated sedation programs.

Currently, the sedation training for non-anesthesiologists is variable and institution-dependent [3]. Despite a detailed description of clinical practice guidelines provided by the American Society of Anesthesiologists task force on sedation and analgesia, there is much room to improve sedation education for non-anesthesiologists [4]. In a commentary by Toney et al., the authors emphasized the need to incorporate robust sedation training in pediatric hospital medicine fellowship curricula [5]. Similarly, Sulton et al. identified notable delays in critical task performance during simulated pediatric airway events in emergency medicine training [6]. Given the need, we created a virtual reality (VR) pediatric sedation curriculum for students, residents, fellows, and attending physicians.

Our hospital offers a two-week pediatric sedation and analgesia elective rotation for trainees. The program entails the planning of sedation and analgesia, medication understanding, hands-on monitoring for adverse events, and proficiency in airway assessment and management. While adverse events during procedural sedation are relatively uncommon, infants and children are a vulnerable population due to their unique airway anatomy, cardiorespiratory physiology, and response to sedation-analgesia medications. Due to the rarity of these events and the perceived need and feedback to improve learners’ experience in adverse event training, we identified a gap in providing a comprehensive experience in this area and utilized simulation to improve our education.

Simulation has been recognized as a key teaching modality to learn high-stakes, low-frequency events [7-9]. In the realm of simulation, VR has expanded as a robust, immersive experience that helps learners have one-on-one, self-paced learning. Leveraging state-of-the-art VR education software from Enduvo Inc. (Peoria, USA), we developed training around adverse and low-frequency events within pediatric sedation that require stepwise learning for stabilization. Our VR curriculum consists of four competency-based training modules, including managing three adverse events, specifically apnea due to airway obstruction, laryngospasm, and chest wall rigidity, and a learning module on nitrous oxide use. The study aims to develop a simulation-based curriculum for all sedation learners, ensuring a comprehensive learning experience.

## Materials and methods

Study design

This study was conducted at OSF Healthcare St. Francis Medical Center and OSF HealthCare Children's Hospital of Illinois in the Midwest region of the United States. The Peoria Institutional Review Board issued approval number 2130903-1. Our institution has been running a well-established sedation program for over 20 years. An elective rotation of two weeks is offered to interested students, residents, and fellows, and approximately 18-20 learners take part in the sedation elective each year. To the existing two-week sedation elective program, a two-hour VR course was added as part of their rotation. This cross-sectional study aims to evaluate the experience of trainees using VR for rare yet crucial aspects of pediatric sedation, specifically those related to the management of adverse events and the setup of the inhaled nitrous oxide machine.

The sedation training was created by subject matter experts, who utilized HTC Vive hardware with locally developed Enduvo software from Peoria, USA, for content creation. The course was divided into four modules, three of which focus on troubleshooting airway complications such as apnea, laryngospasm, and chest wall rigidity caused by the commonly used sedatives. The fourth module provided education on the setup and use of inhaled nitrous oxide. The training took place in a VR environment within a designated simulation space. The experts meticulously developed the curriculum to ensure it is effective and comprehensive.

Objectives

The learning objectives of the curricula were the following:

1. To identify uses, indications, and complications of the sedative agents used in pediatrics, including propofol, benzodiazepines, and opiates.

2. To recognize and troubleshoot life-threatening situations during procedural sedations, including apnea, laryngospasm, and chest wall rigidity.

3. To describe the use of inhaled nitrous oxide equipment and review its setup and troubleshooting mechanisms.

Each module was 20 minutes in length. After the initial instructional segment, each module was subdivided into three segments: an introduction to the concept, a detailed explanation of relevant physiology and anatomy, and techniques for managing these events. The segments included a variety of multimedia elements such as videos and three-dimensional images, which are also referred to as assets. Learners were expected to complete embedded quizzes after finishing each segment to reinforce their understanding. These modules were designed to be brief and engaging while allowing learners to take breaks if needed. On completion of the 15 minutes, a five-minute pre-recorded summary was provided for a quick review. Learners were able to complete the tutorial at their own pace and repeat it as many times as needed until they felt comfortable.

Study population

All learners who rotated through the sedation elective from 2019 to 2022 were expected to complete the VR modules as part of their elective. Residents experiencing motion sickness were exempt from completing the modules if they had any concerns. After finishing all the modules, participants were invited to complete a three-point Likert scale survey to assess their perceptions, knowledge acquisition, and confidence levels regarding the VR curriculum (Appendix). The survey results were anonymized to prevent expectation bias.

## Results

Thirty-five participants were included in the study: seven medical students, 22 resident physicians, four pediatric hospital medicine fellows, and two attending physicians. Ninety-seven percent (34/35) of the learners had zero to five years of clinical experience. Overall, 16 (45.7%) participants had prior pediatric sedation/anesthesia training, which included any training as a medical school rotation, Society for Pediatric Sedation (SPS) course, computer-based course, sedation or anesthesia elective, or didactic training on adverse events. Of all the participants, 17 (48%) had experienced adverse events during sedation (Table 1).

**Table 1:** Participant characteristics and experience with sedation-related events SPS: Society for Pediatric Sedation

Participant characteristics	N (%)
Years of clinical experience	
0-5 years	34 (97)
6-10 years	1 (3)
Formal sedation training received (Includes medical school rotation, SPS Course, computer-based course, sedation or anesthesia elective, didactic training on adverse events (Y)	16 (45.7)
Adverse events experienced (Y)	17 (48)
Total participants	35 (100)
Category	
Medical students	7 (20)
Emergency medicine residents	7 (20)
Pediatric residents	6 (17.1)
Internal medicine-pediatrics	5 (14.3)
Other(s) residents	4 (11.4)
Fellows	4 (11.4)
Faculty	2 (5.7)

For the apnea module, 17 (60%) individuals felt adequately prepared with VR training, while 11 (39%) reported an equivalent level of preparation compared to their previous methods. Confidence levels in apnea management were high, with nine (33%) reporting complete confidence, 18 (66%) expressing some confidence, and no respondents reporting a lack of confidence. In the laryngospasm module, 20 (95%) respondents expressed feeling significantly more well-prepared through VR training. Conversely, one (5%) reported an equivalent level of readiness compared to their previous training (Table 2).

**Table 2:** Summary of learners' self-reported preparedness and confidence levels following the completion of modules VR: virtual reality

Self-preparedness	VR prepared me better than prior training methods N (%)	VR gave me the same level of preparation as my prior training N (%)	VR did not prepare me as well N (%)	Not applicable N (%)
Nitrous oxide	18 (75)	5 (20)	1 (4.16)	11 (31.4)
Chest wall rigidity	16 (84)	3 (15)	0 (0)	16 (45.7)
Apnea	17 (60)	11 (39)	0 (0)	7 (20)
Laryngospasm	20 (95)	1 (5)	0 (0)	14 (40)
Confidence	100% (completely) confident	50% (somewhat) confident	Not confident	Not applicable
Nitrous oxide	15 (45)	13 (39)	5 (15)	2 (5.7)
Chest wall rigidity	3 (17)	13 (76)	1 (5.8)	18 (51.4)
Apnea	9 (33)	18 (66)	0 (0)	8 (22.9)
Laryngospasm	5 (27)	12 (66.6)	1 (5.5)	17 (48.6)

Confidence levels in laryngospasm management were more varied, with five (27%) expressing complete confidence, 12 (66.6%) reporting some confidence, and one (5.5%) indicating a lack of confidence.

For the chest wall rigidity module, a substantial 16 (84%) participants reported a high level of self-preparedness through VR training, and five (15%) reported an equivalent preparation level as their prior training. Importantly, none of the respondents reported feeling unprepared with VR in this scenario. Within the same module, three (17%) reported complete confidence, 13 (76%) expressed some confidence, and one (5.8%) lacked confidence (Table 2).

For the nitrous oxide setup, a majority of respondents, totaling 18 (75%), conveyed a sense of readiness attributed to VR training, while an additional five (20%) indicated an equivalent level of preparation compared to their prior training methods. Notably, one (4.16%) expressed dissatisfaction, feeling that VR did not adequately prepare them, and 11 (31.4%) responders did not complete that module. Confidence levels in the context of nitrous oxide were more diverse, with 15 (45%) expressing complete confidence, 13 (39%) reporting some confidence, and five (15%) indicating a lack of confidence (Table 2).

Learners were also requested to complete a post-survey regarding the relevance and effectiveness of VR technology (Table 3).

**Table 3:** Feedback from learners on the relevance and effectiveness of VR technology VR: virtual reality

Participants’ feedback on relevance and difficulty of VR technology	N (%)				
Relevance of VR to current clinical role	Extremely relevant	Very relevant	Relevant	Neutral	Irrelevant
	12 (34)	14 (40)	7 (20)	2 (5.7)	0 (0)
Level of difficulty with technology (head-mounted display, hand tools, software	Very simple	Somewhat simple	Neutral	Somewhat difficult	Very difficult
	19 (54)	11 (31)	3 (8.5)	2 (5.7)	0 (0)

A significant portion of learners find VR to be relevant to their clinical roles, with a majority rating it as either extremely or very relevant. Additionally, the majority of learners reported that the technology, including head-mounted displays, hand tools, and software, was perceived to be very simple to use. These findings suggest a positive outlook on the incorporation of VR technology in the clinical learning environment, with a notable emphasis on its relevance and user-friendly nature.

## Discussion

Our findings indicate that the VR training was well received by the participants and improved their confidence and self-preparedness to handle adverse reactions as compared to prior educational training modalities. The participants showed a positive attitude toward integrating VR technology into sedation curricula. This pilot study highlights the potential benefits of incorporating such technology in the training of healthcare professionals.

VR has emerged as an innovative educational tool, demonstrating effectiveness in improving manual and immediate decision-making skills [10]. VR has been studied specifically in surgical and emergency medicine specialties, where clinical skills training is needed to improve clinical outcomes [11-13].

We applied the same concept in developing a pediatric sedation curriculum to lay the groundwork for understanding the physiological and anatomical factors crucial for mastering airway complications through 3D visualization. By creating a less stressful learning environment with an individualized and self-paced focus, learners are better prepared for real-life situations. The interactivity of the video and embedded quizzes helps learners assimilate the information better because they provide better visual guidance as compared to traditional learning.

**Figure 1:**
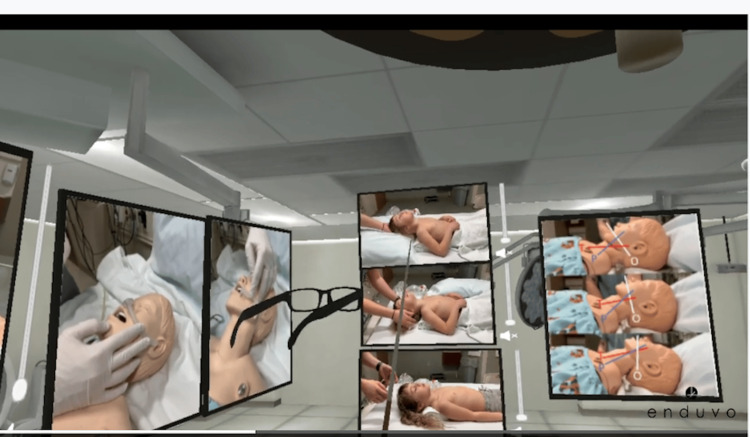
Virtual reality image of apnea module Credit: This figure is a snapshot of one of the modules in the lecture series created by Enduvo, Inc. Each manikin was utilized from the Jump Trading Simulation Center at OSF Saint Francis Medical Center, and the photograph was taken by the content developers at Enduvo, Inc. Written informed consent has been provided by the patient to have the case details and any accompanying images published.

**Figure 2:**
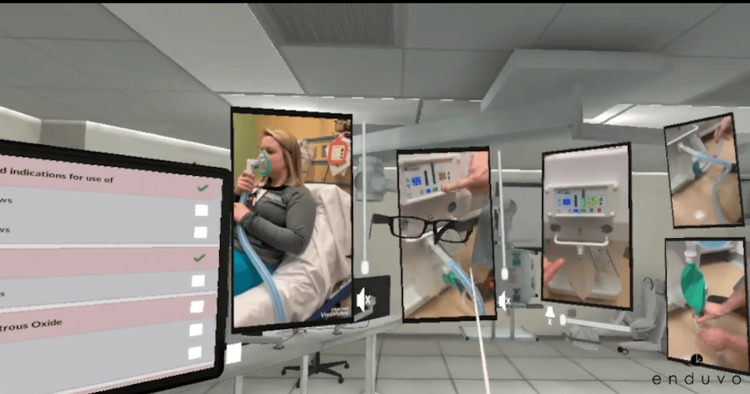
Virtual reality image of nitrous oxide module Credit: This figure is a snapshot of one of the modules in the lecture series created by Enduvo, Inc. Each manikin was utilized from the Jump Trading Simulation Center at OSF Saint Francis Medical Center, and the photograph was taken by the content developers at Enduvo, Inc. Written informed consent has been provided by the patient to have the case details and any accompanying images published.

The rapid growth of pediatric sedation services nationally merits more opportunities for residents and fellows in the future years. A study by Kamat et al. demonstrated a steep uptrend in the sedations being performed by non-anesthesiologists over the past decade [1]. As a result, there is a growing need for residents and fellows to receive additional training in this specialty, particularly with the emergence of a pediatric hospital medicine fellowship. While the SPS is a national consortium and provides simulation-based training for pediatric physicians in sedation, these courses are only offered at limited times throughout the year. Introducing VR training for sedation will provide young physicians and trainees with a more focused and intensive learning experience while prioritizing the safety of real patients (Figures 1-2).

The pediatrics sedation research consortium reported that the most common serious adverse events involve laryngospasm and airway obstruction [14]. While chest wall rigidity is exceedingly rare, managing it effectively can be challenging without receiving additional specialized training. Moreover, proficiency in handling nitrous oxide necessitates hands-on training for practitioners to feel at ease with the equipment. A lack of familiarity with the equipment could pose a barrier to its effective utilization. Competence to use the equipment is achieved after repeated practice, which is not feasible for learners with patients and families. These findings influenced our decision to select them for our training program and integrate VR training into our program to provide a solution to enhance familiarity and competence among practitioners. Management of these complications requires learning specific maneuvers. VR enhanced the learner’s experience by providing education on anatomic variances, adjustment techniques, and troubleshooting methods in a controlled environment, enabling them to evaluate each step and refine their skills before applying them in real patient scenarios. It also allows access to a 3D, immersive setting that contains the equipment and replicates the environment they are being asked to work in.

Our study had a few limitations. One limitation is that there was a heterogeneous sample of participants, meaning that learning objectives and outcomes may differ for each group. The purpose of this study was not to prove the effectiveness of VR technology in medical education. Future research can consider comparing traditional video recording or other educational modalities with VR experience to compare efficacy. While VR is an excellent educational tool to utilize, some participants experienced nausea and dizziness after using VR headsets, highlighting a barrier that can limit its use. All immersive pieces of training, such as augmented reality and mixed reality headsets, can cause the same symptoms, although the newest technologies are minimizing these effects by switching modes that are most stable.

## Conclusions

Our study confirms heightened confidence levels and self-preparedness among participants after undergoing VR training for complex sedation events. Future projects should explore the potential impact of VR training on the clinical implications of these learned skills. This will pave the way for further advancements in medical education.

## Appendices

1.     What is your current clinical role/position/primary employment?

a.     Registered Nurse

b.     Physician-Emergency Medicine attending

c.     Physician-Pediatric attending

d.     Physician-Other (please specify: ____________________________)

e.     Resident Physician

f.      Medical student

g.     Other (Please specify: ____________________________________)

2.     How many years of clinical experience do you have?

a.     0-5 years

b.     6-10 years

c.     11-20 years

d.     21-30 years

e.     31+ years

3.     If your specialty is Pediatrics, what is your primary role or practice setting?

a.     Pediatric subspecialty (please specify: ___________________)

b.     Pediatric hospitalist-not on sedation team

c.     Pediatric hospitalist-on sedation team

d.     Other (please specify: ________________________)

4.     A. Have you ever completed formal training in management of sedation or anesthesia?

a.     Yes

b.     No

B. If answer to above is YES:  (If no, skip to #5)

What type of training did you complete (circle all that apply)

c.     Didactic training on sedation adverse events

d.     A rotation in pediatric sedation  

e.     Computer-based module/online course

f.      Article in a journal (online or print)

g.     SPS Sedation course

h.     Other (Please specify: ______________________________________)

Please identify which modules you completed today:

a.     Nitrous Oxide sedation (if yes, go to the next Q and skip Qs 6,7 & 8)

b.     Apnea and sedation monitoring (if yes, skip to Q6)

c.     Chest Wall Rigidity (if yes, skip to Q7)

d.     Laryngospasm (if yes, skip to Q8)

5.     Compared to your prior training in management of Nitrous Oxide use, how well did Enduvo prepare you to manage this condition?

a.     Enduvo module did not prepare me as well

b.     Enduvo module gave me the same level of preparation as my prior training

c.     Enduvo module prepared me better than prior training methods

6.     Compared to your prior training in management of apnea and sedation monitoring, how well did Enduvo prepare you to manage this condition?

a.     Enduvo module did not prepare me as well

b.     Enduvo module gave me the same level of preparation as my prior training

c.     Enduvo module prepared me better than prior training methods

7.     Compared to your prior training in management of chest wall rigidity, how well did Enduvo prepare you to manage this condition?

a.     Enduvo module did not prepare me as well

b.     Enduvo module gave me the same level of preparation as my prior training

c.     Enduvo module prepared me better than prior training methods

8.     Compared to your prior training in management of laryngospasm monitoring, how well did Enduvo prepare you to manage this condition?

a.     Enduvo module did not prepare me as well

b.     Enduvo module gave me the same level of preparation as my prior training

c.     Enduvo module prepared me better than prior training methods

9.     Have you ever experienced any of the adverse events in a patient during administration of procedural sedation or anesthesia?

a.     Yes

b.     No

10.  During which procedure did you happen to experience an adverse event/which event did you experience?

a.     Nitrous Oxide sedation

b.     Apnea and sedation monitoring

c.     Laryngospasm

d.     Chest Wall Rigidity

Learning

11.  Please indicate your percent confidence level in managing this type of patient or performing this skill BEFORE your participation in the Enduvo sedation module:

0% (Not Confident)         50% (Somewhat Confident)        100%(Completely Confident)

12.  Please indicate your percent confidence level in managing this type of patient or performing this skill AFTER your participation in the Enduvo sedation module:

0% (Not Confident)        50% (Somewhat Confident)        100% (Completely Confident)

13.  After completing the Enduvo MCI triage module, how confident are you in the ability to manage laryngospasm?

1. I would require complete hands-on guidance

2. I am able to manage laryngospasm, but require constant direction

3. I can have some independence but would require intermittent direction

4. I could have independence but still require supervision for safe practice

5. I could function with complete independence, understands risk and performs safely, practice ready

Reaction

14.  Please rate the relevance of the Enduvo sedation module to your CURRENT clinical role. (Likert Rating)

a.     Extremely Irrelevant / Detrimental

b.     Very Irrelevant / Detrimental

c.     Irrelevant / Detrimental

d.     Neutral

e.     Relevant / Outstanding

f.      Very Relevant / Outstanding

g.     Extremely Relevant / Outstanding

15.  How difficult was the technology (head-mounted display, hand tools, software) to use?

a.     very difficult

b.     somewhat difficult

c.     neutral

d.     somewhat simple

e.     very simple

16.  Did you note any information that you felt was inaccurate, unclear, or needed additional coverage? (you can also email this information to Dr. Riech or Dr. Shaikh)

17.  Any other feedback on your training experience today?

## References

[1] Kamat PP, Sulton C, Kudchadkar SR (2019). Procedural sedation outside the operating room and potential neurotoxicity: analysis of an at-risk pediatric population. Acad Pediatr.

[2] Turmelle M, Moscoso LM, Hamlin KP, Daud YN, Carlson DW (2012). Development of a pediatric hospitalist sedation service: training and implementation. J Hosp Med.

[3] Miller AF, Monuteaux MC, Bourgeois FT, Fleegler EW (2018). Variation in pediatric procedural sedations across children's hospital emergency departments. Hosp Pediatr.

[4] (2002). Practice guidelines for sedation and analgesia by non-anesthesiologists. Anesthesiology.

[5] Toney M, Pattishall S, Garber M (2020). The time Is now: standardized sedation training for pediatric hospitalists. Pediatrics.

[6] Sulton CD, Burger RK, Figueroa J, Taylor TR (2021). Evaluation of pediatric procedural sedation education in pediatric emergency medicine fellowships. Medicine (Baltimore).

[7] Smallman B (2002). Pediatric sedation: can it be safely performed by non-anesthesiologists?. Curr Opin Anaesthesiol.

[8] Schinasi DA, Nadel FM, Hales R, Boswinkel JP, Donoghue AJ (2013). Assessing pediatric residents' clinical performance in procedural sedation: a simulation-based needs assessment. Pediatr Emerg Care.

[9] Eppich WJ, Nypaver MM, Mahajan P, Denmark KT, Kennedy C, Joseph MM, Kim I (2013). The role of high fidelity simulation in training pediatric emergency medicine fellows in the United States and Canada. Pediatr Emerg Care.

[10] Bracq MS, Michinov E, Arnaldi B, Caillaud B, Gibaud B, Gouranton V, Jannin P (2019). Learning procedural skills with a virtual reality simulator: an acceptability study. Nurse Educ Today.

[11] Lee GI, Lee MR (2018). Can a virtual reality surgical simulation training provide a self-driven and mentor-free skills learning? Investigation of the practical influence of the performance metrics from the virtual reality robotic surgery simulator on the skill learning and associated cognitive workloads. Surg Endosc.

[12] Radi I, Tellez JC, Alterio RE (2022). Feasibility, effectiveness and transferability of a novel mastery-based virtual reality robotic training platform for general surgery residents. Surg Endosc.

[13] Abbas JR, Chu MM, Jeyarajah C (2023). Virtual reality in simulation-based emergency skills training: a systematic review with a narrative synthesis. Resusc Plus.

[14] Kamat PP, McCracken CE, Simon HK, Stormorken A, Mallory M, Chumpitazi CE, Cravero JP (2020). Trends in outpatient procedural sedation: 2007-2018. Pediatrics.

